# Supplementary material for: Model-based cell clustering and population tracking for time-series flow cytometry data
Source: BMC Bioinformatics. 2019 Dec 27;20(Suppl 23):633. doi: 10.1186/s12859-019-3294-3 (PMC6933651; doi:10.1186/s12859-019-3294-3)
Supplement: Supplementary file 1 — Additional file 1 Supplementary Figures. [file 12859_2019_3294_MOESM1_ESM.docx]

**Supplementary Materials for**

**Model-based cell clustering and population tracking for time-series flow cytometry data**

Kodai Minoura^1,2^, Ko Abe^1^, Yuka Maeda^3^, Hiroyoshi Nishikawa^2,3^, Teppei Shimamura^1,*^

^1^Division of Systems Biology, ^2^Division of Immunology, Graduate School of Medicine, Nagoya University, Nagoya, Japan. ^3^Division of Cancer Immunology, Research Institute/EPOC, National Cancer Center, Tokyo/Chiba, Japan.

*To whom correspondence should be addressed: shimamura@med.nagoya-u.ac.jp

**a**

**b**

**Supplementary Figure 2 Elbow plot for real world data.**

a. Elbow plot for Landrigan’s study. The number of clusters was determined to 16. b. Elbow plot for Huang’s study. The number of clusters was determined to 26.

**Supplementary Figure 1 Elbow plot for simulation study.**

Cluster size 2 to 8 was tested for every repeat of simulation. The elbow plot shows SSE for 10 repetition. Note that SSE sharply decrease between cluster size 2 and 3, and only moderately decrease thereafter. Error bars are standard error.

**Supplementary Figure 3 Multivariate Gaussian mixture distribution approximates flowcytometry data.**

Histograms of marker intensities for 16 clusters identified in Landrigan’s study were plotted. Mean and variance were calculated for each histogram to overlay Gaussian density curves.
